# Analysis of Fatty Acids and Amino Acids of Three Local Freshwater Bagridae Fish Species in the Kampar Kanan River, Indonesia, for Food Security

**DOI:** 10.1155/2024/6639837

**Published:** 2024-01-04

**Authors:** Azrita Azrita, Hafrijal Syandri, HazlinaAhamad Zakeri, Harfiandri Damanhuri, Netti Aryani

**Affiliations:** ^1^Faculty of Fisheries and Marine Science, Bung Hatta University, 25131 Padang, West Sumatra Province, Indonesia; ^2^Faculty of Science and Marine Environment, Universiti Malaysia Terengganu, Malaysia; ^3^Faculty of Fisheries and Marine Science, Riau University, 28293 Pekanbaru, Riau Province, Indonesia

## Abstract

Fish have become an irreplaceable dietary source of animal protein, especially among households with low socioeconomic status in rural and urban areas of Indonesia. This study is aimed at analysing the proximate composition, minerals, fatty acids, and amino acids of three local Bagridae fish species in the Kampar Kanan river, Indonesia. The standard AOAC method was employed to examine the proximate composition of the carcass, and the analysis of amino acids and fatty acids was conducted through HPLC and GC-MS, respectively. The mineral content was determined using AAS. The nutrient composition results of *Hemibagrus nemurus*, *Hemibagrus wyckii*, and *Mystus nigriceps* revealed that the protein content was 24.26%, 22.57%, and 21.39% (% dry weight), whereas the total lipid content was 6.64%, 7.47%, and 7.75%, respectively. Regarding mineral contents, the calcium levels ranged from 1.49 to 1.66 mg/g, iron levels from 28.35 to 40.36 *μ*g/g, and zinc levels from 24.03 to 54.46 *μ*g/g. Among the fatty acids, palmitic acid was the most abundant in all three species, accounting for 25.59–30.70% of the total fatty acids. Additionally, significant amounts of C18:1 (oleic acid), C18:0 (stearic acid), and C20:4 (arachidonic acid) were also detected as primary fatty acids. The calculated atherogenic index values in the three species of Bagridae fish ranged from 0.73 to 0.99, while the thrombogenic index values varied between 0.54 and 0.75. The predominant amino acids found in the three species of Bagridae fish were glutamic acid with their concentrations ranging from 9.10 to 24.34%. These results indicate that consuming the meat of these three freshwater Bagridae fish species caught in the wild does not pose any health risks to consumers. They can be considered a safe and suitable food source with good nutritional quality.

## 1. Introduction

Fish serve as a high-quality protein source that is essential for satisfying dietary protein requirements, especially in areas where other protein sources are few or difficult to obtain [1, 2]. Their accessibility can aid in ensuring a steady supply of food, particularly for communities whose main source of income is fishing or aquaculture [3]. Fish can contribute to a more varied selection of protein sources in communities, resulting in a diet that is more nutritious and balanced. Dietary fish can treat nutrient deficits and enhance general nutritional health. Fish are generally considered safe and offer a lower risk of contamination with harmful substances compared to some land-based animal products. A reliable indicator of a fish's quality, nutritional value, physiological status, and environment is its chemical makeup, which is found in fish flesh [4].

*Hemibagrus nemurus*, locally known as “baung,” *Hemibagrus wyckii*, known as “geso,” and *Mystus nigriceps*, known as “ingir-ingir,” are three species of catfish found in the Kampar Kanan River, Indonesia [5, 6]. Due to their high market value, these species have been identified as significant economic resources for rural communities [7]. Therefore, the analysis of the nutritional quality of fish meat in the study area is very important to determine whether it is beneficial to human health.

Knowing the composition of fatty acids and amino acids in fish meat is an essential factor that should not be ignored [8]. This information provides valuable insight into the nutritional quality of fish and the value of more nutritious and healthy foods [9]. In addition, a better understanding of the composition of fatty acids and amino acids can assist in selecting a suitable feed [10], developing a balanced diet [11], and maintaining optimal fish meat nutrition [12].

Omega-3 polyunsaturated fatty acids (PUFAs), particularly docosahexaenoic acid (DHA) and eicosapentaenoic acid (EPA) derived from fish oil, exhibit anti-inflammatory and antigastrointestinal cancer properties, making them crucial as immune-boosting nutrients [13, 14]. Studies have demonstrated that *ω*-3 and *ω*-6 PUFAs exhibit beneficial effects in the management of cardiovascular diseases and cancers [15, 16]. The fatty acid composition of PUFAs can differ among different types of fish, including both freshwater and marine species [17, 18]. Amino acids (AAs), such as cysteine, arginine, tyrosine, glycine, proline, and serine, play a vital role in illness and stress situations and in the prevention of inflammation and repair processes in fish intestines [19, 20].

Furthermore, knowledge of the atherogenicity index (AI) and thrombogenicity index (TI) of fatty acids in the three local freshwater fish species of Bagridae can contribute to improving dietary guidelines. The AI and TI can be used to develop recommendations aimed at improving heart health [21, 22]. These recommendations may involve selecting fish with favourable fatty acid profiles, allowing individuals to choose the most suitable fish species based on their specific needs [17, 23]. Therefore, this study is aimed at examining the nutritional composition profile, mineral content, amino acids, fatty acids, and AI and TI of three freshwater fish species, namely, *H. nemurus*, *H. wyckii*, and *M. nigriceps*. These three fish species are generally consumed by people who live around the Kampar Kanan River in Kampar Regency, Riau Province, Indonesia.

## 2. Material and Methods

### 2.1. Animal Material

**Figure 1:**
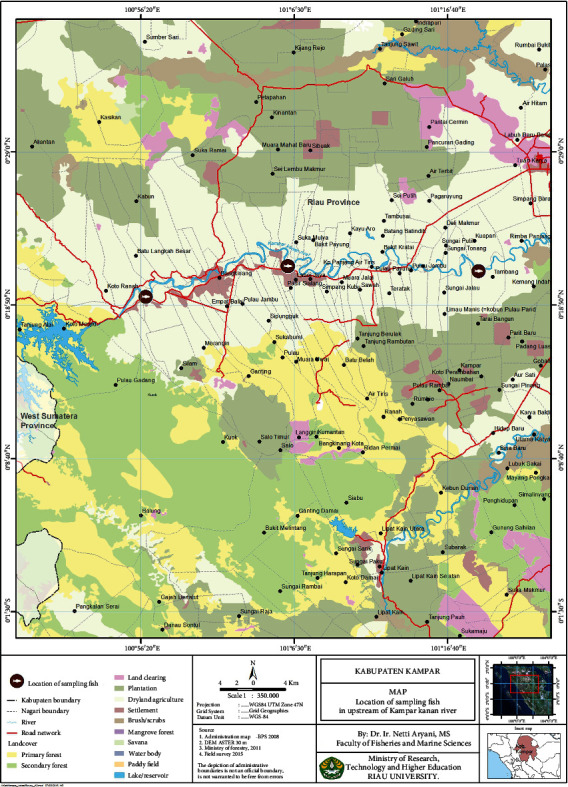
Map of Kampar Regency, Riau Province, and sampling locations of the three species of Bagridae fish.

In this study, a total of 27 Bagridae fish belonging to three distinct species (*H. nemurus*, *H. wyckii*, and *M. nigriceps*) were examined. The specimens were obtained from local fishers operating in the upper stretches of the Kampar Kanan River in Kampar Regency, Riau Province (Figure 1). Sampling took place between June and August 2022 and was verified by the Fisheries Department, Faculty of Fisheries and Marine Science, Bung Hatta University, Indonesia. The samples were placed in plastic bags and transported to the laboratory in an insulated icebox. The samples were then grouped based on their respective sampling locations.

### 2.2. Biometry Measurement

Upon reaching the laboratory within 10 hours, the fish samples were subjected to gutting and washing. Each specimen was weighed (TW) and measured to determine the standard length (SL) as well as the depth or maximum height (H). TW was measured using a balance scale (OHAUS Model CT 6000 USA) with a precision of 0.01 g. The length measurement was taken from the tip of the mouth to the end of the upper lobe of the caudal fin, representing the total body length, using a metre ruler with an accuracy of 1 mm. The height measurement involved a vertical assessment of the body's maximum height measured using a Digital Sekhmet Sigma Vernier calliper with an accuracy of 1 mm. The condition factor (CF) was calculated using the formula CF = (TW/SL^3^) × 100.

### 2.3. Proximate, Amino Acid, and Mineral Content Analysis

The proximate carcass composition was assessed following the AOAC standard methods [24]. The samples were dried to a constant weight at 105°C. The crude protein content was analysed using the standard Kjeldahl method, which involves multiplying the nitrogen content by a factor of 6.25. The Soxhlet method with ether extraction was employed to analyse crude lipids. The ash content was obtained by incinerating the samples at 550°C for 16 h. The amino acid composition was determined using a high-performance liquid chromatography (HPLC) system, which consisted of a Waters® 1525 binary HPLC pump, Waters® 717 autosamplers, and Waters® 2475 multi-*λ* fluorescence detector (with excitation at 250 nm and emission at 395 nm). The samples were hydrolysed in triplicate with 6 N hydrochloric acid for 24 h at 11°C [25].

For mineral data composition (Na, Mg, Ca, K, P, Fe, Cu, Mn, and Zn), the ashed feed sample and carcass were dissolved in 1 ml of hydrochloric acid (35% *v*/*v* Suprapur®, Merck) and then filtered with cellulose filter paper (Watchman No. 1, International Ltd; Maidstone, UK) and diluted to an appropriate concentration for each elemental mineral. P levels were analysed with a Perkin-Elmer AA spectrophotometer mod 3110 (Norwalk, CT, USA).

### 2.4. Fatty Acid Analysis

The fish meat was examined utilizing the gas chromatography-mass spectrometry (GC-MS) method. The method of Folch et al. (1957) modified by Rajion et al. [26] was employed to perform the total fat extraction. This involved using a solvent system consisting of chloroform and methanol at a 2 : 1 (*v*/*v*) ratio. The process of transmethylation was conducted using a solution of 14% methanolic boron trifluoride. The fatty acid composition of the meat was then analysed at the SIG Laboratory, Accredited Testing Laboratory-LP-184-IDN.

The nutritional quality of lipids (AI and TI) was calculated using the following equations [27]:

(1) $$\begin{array}{c} \text{AI}=\frac{\left[\left(\text{C}12:0+4\times \text{C}14:0+\text{C}16:0\right)\right]}{\left[\left(\sum \text{M}\text{UFA}+\sum n-6\right)+\sum n-3\right]},\\ \text{TI}=\frac{\left[\left(\text{C}14:0+\text{C}16:0\right)\right]}{\left[\left(0.5\times \sum \text{M}\text{UFA}\right)+\left(0.5\times \sum n-6\right)+\left(3\times \sum n-3\right)+\left(\sum n-3/\sum n-6\right)\right]}, \end{array}$$

where AI is the atherogenic index, TI is the thrombogenic index, C12 : 0 is the lauric acid, C14 : 0 is the myristic acid, C16 : 0 is the palmitic acid, ∑MUFA is the sum of the concentrations of all monosaturated fatty acids, ∑*n* − 6 is the sum of the concentrations of n-6 polyunsaturated fatty acids, and ∑*n* − 3 is the sum of the concentrations of n-3 polyunsaturated fatty acids.

### 2.5. Data Analysis

Data analysis was performed using the SPSS 16.0 software package (SPSS; Chicago, IL). The homogeneity of variances was assessed using Levene's test. One-way ANOVA was carried out to determine the parameters of proximate and amino acid composition, including the composition of fatty acids and nutritional quality of lipids (AI and TI) for each species, followed by the post hoc Duncan's multiple range test [28]. The results are reported as the mean *values* ± *standard* errors for each parameter.

## 3. Results

Table 1 presents the average wet weight, standard length, height, and the results of meat nutritional composition analysis for three indigenous freshwater Bagridae species in the Kampar Kanan river, Indonesian. The moisture content (% wet weight) ranged from 82.40 to 85.39%. Among the species examined, *M. nigriceps* exhibited the lowest protein content at 21.39%, while *H. nemurus* displayed the highest value at 24.26%. However, *M. nigriceps* had a higher mineral content, including iron and zinc.

Table 2 summarizes the fatty acid composition (% total fatty acid) for the three Bagridae fish species. C16:0 was the predominant fatty acid in the three species of Bagridae fish, with percentages ranging from 25.09 to 30.71%. The additional prominent fatty acids found were C18: 1, C18:0, and C22:6. *H. nemurus* exhibited a *ω*-6/*ω*-3 ratio below 1 (0.82), while *H. wyckii* and *M. nigriceps* had ratios of 1.11 and 1.02, respectively. The obtained PUFA/saturated fatty acid (SFA) (P/S) ratio varied between 0.61 and 0.76. The AI values varied between 0.76 and 0.99 and the TI between 0.40 and 0.63 (Table 3).

Table 4 presents the amino acid composition (% of total protein) of the three Bagridae species. Glutamic acid was the dominant amino acid, ranging from 19.29 to 24.34%, followed by aspartic acid, which ranged from 9.21 to 11.27%. The lysine content was consistent, ranging from 9.67 to 9.87%. In *H. nemurus*, the levels of various amino acids ranged from 0.86 to 24.35%. Similarly, in *H. wyckii*, the amino acid levels ranged from 0.76 to 21.58%, while in *M. nigriceps*, the levels ranged from 0.74 to 19.29%.

## 4. Discussion

The results of this study provide important new information about the nutritional makeup of the studied species of Bagridae fish. The biometric measures of the fish yielded important information about their physical traits and health. Researchers are able to determine the average weight of fish at various lengths by examining the link between length and weight for a particular species. This knowledge is essential for understanding the trends in fish population growth, identifying potential shifts in growth rates over time, and assessing the general health and welfare of fish [29]. A variety of factors, including sex, age, maturity level, size, stomach fullness level, sampling strategies, sample sizes, and environmental circumstances, have an impact on fish health and the parameters that determine length-weight connections in fish [30]. However, none of these factors were considered in our study. In general, according to the theory put forth by Bagenal and Tesch [31], heavier fish of a given length are in better physiological condition. The condition factor is also used in fishery science to compare the health, fatness, and condition of fish [32]. A condition factor of >1 is favourable, indicating a good feed nutrition level and suitable living habitat [33]. Thus, based on the findings in this study, the Kampar Kanan River provides a suitable habitat for the fish, ensuring their accessibility and availability for community consumption.

The fish samples exhibited good nutritional profiles, with high amounts of proteins and lipids as well as moisture content contributing to their overall nutritional value, according to the results of proximate analysis and mineral composition (see Table 1). These results imply that the fish species studied provide a possible source of nutrients to solve issues with food security. The study of the mineral composition revealed the existence of critical minerals such as phosphorus (P), calcium (Ca), magnesium (Mg), iron (Fe), and zinc (Zn) in the fish samples in addition to proteins and lipids. All three Bagridae fish significantly differed from each other in terms of their crude ash content and mineral profile (see Table 1). However, in general, P and Ca are the two highest minerals found in all fish. Both of these macrominerals are good for human bone and tooth health, as key components of hydroxyapatite, the mineral matrix that provides strength and structure to bones and teeth [34].

In the current study, the meat of three species of Bagridae fish from the Kampar Kanan River in Indonesia was found to contain an abundance of microminerals such as Fe and Zn. *M. nigriceps* has been reported to exhibit higher mineral content, including Fe and Zn. This species is categorized as a small indigenous fish (SIF) with a maximum standard length of approximately 10.29 cm. According to [35], SIFs are generally known to be rich in Ca, Fe, and Mn. On the other hand, scale flour from three species of freshwater fish, such as *Osphronemus goramy*, *Cyprinus carpio*, and *Oreochromis niloticus*, also contains high levels of macrominerals and microminerals [36].

In this study, all three Bagridae fish had a total fat content of approximately 7–8% by weight, with a higher water content ranging from 82.40 to 85.39% (Table 1). These values are similar to those found in wild and cultured sea bass *Dicentrarchus labrax* [37]. Additionally, the fish species can be grouped into different categories, including high-fat fish (>8%), medium-fat fish (4–8%), low-fat fish (2–4%), and lean fish (<2%) [38]. According to the classification established by [38], the three indigenous Bagridae fish species are characterized as having a moderate fat content level, which is reflected by the high levels of DHA and EPA in the meat of *H. nemurus*, *H. wyckii*, and *M*. *nigriceps* (Table 2). Therefore, we recommend that consuming the meat of these three fish species can indirectly boost food security in rural and urban communities.

All three Bagridae fish species analysed in the study contained arachidonic acid (C20:4), at 12.13% for *H. nemurus*, 12.49% for *H. wyckii*, and 12.41% for *M. nigriceps*. Arachidonic acid serves as a precursor for synthesizing prostaglandins and leukotrienes [39]. Leukotrienes are crucial in allergic responses, inducing smooth muscle contraction and increasing vascular permeability [40]. Additionally, all three species also contained EPA ranging from 1.35 to 2.34% and DHA ranging from 11.56 to 15.65%. The increased consumption of omega-3 fatty acids, such as EPA and DHA, has been linked to a decreased risk of pain development in older individuals and to the prevention of Alzheimer's disease [41, 42].

As determined by the current study, the *ω*-6/*ω*-3 ratios of the three Bagridae fish species ranged from 1 : 0.8 to 1 : 0.97. It was found that all three fish species had *ω*-6/*ω*-3 ratios within the recommended range for a typical Indonesian diet. In contrast, a typical Western diet is characterized by a high *ω*-6/*ω*-3 ratio of 10 : 1 to 20 : 1, while a Mediterranean diet has a ratio of 4 : 1 to 5 : 1 [43]. Additionally, the P/S ratio analysis showed that the three Bagridae fish species are sources of PUFAs that meet the requirements for food safety.

The Food and Agriculture Organization (FAO) and the World Health Organization (WHO) have recommended AI and TI values ranging from 0.4 to 0.5 for promoting human health [44]. In the current study, the observed AI values in the three freshwater species of Bagridae ranged from 0.73 to 0.99, while the TI values varied between 0.40 and 0.63. This finding is associated with a notable disparity in the values of SFAs among the three Bagridae fish species. Based on these findings, we confirm that consuming the meat of the three freshwater species of Bagridae fish caught in the wild does not endanger consumer health and can serve as a suitable source of food safety and nutritional quality.

Fish with a high crude protein content, as shown by the three studied Bagridae fish (Table 1), are regarded as being nutritionally useful for human health. The protein and amino acid contents can directly indicate the nutritional quality of meat [45]. As shown in Table 4, *H. wyckii* had a higher content of essential amino acids (EAAs) than *H. nemurus* and *M. nigriceps*. Among the EAAs, lysine (Lys) was found to be present in the highest amount in all three species. Lys is necessary for tissue growth and repair, reduced stress-induced anxiety, and the conversion of fatty acids into energy [46–48].

More than 60% of the amino acid composition in all three Brigade fish studied comes from nonessential amino acids (NEAAs), particularly glutamic acid (Table 4). Glutamic acid and its amine, glutamine, are highly abundant amino acids found in fish in the free and protein-bound forms [49]. Other amino acids found at abundant levels in the three fish also play important physiological and biochemical functions. The NEAA arginine, for example, is involved in protein synthesis, creatine formation, and various other biochemical processes within the body. The EAAs leucine, phenylalanine, and methionine play important roles in the synthesis of nitric oxide, a molecule that can expand blood vessels [49].

## 5. Conclusions

The present study provides insightful information about the fatty acid and amino acid compositions, biometric measurements, proximate analysis, and mineral composition of three local freshwater species Bagridae fish in the Kampar Kanan river, Indonesian. The findings show their potential as a nutrient-dense, sustainable food source that can support efforts to increase food security. These fish species might be incorporated into the local diet to increase nutrient intake and alleviate nutritional inadequacies, which would ultimately improve Indonesia's overall food security status.

## Figures and Tables

**Table 1:** Results of biometric, proximate, and mineral composition of three species of Bagridae fish.

	Species		
	*Hemibagrusnemurus*	*Hemibagruswyckii*	*Mystusnigriceps*
*Biometric measurements*			
Total weight (g)	389.99 ± 24.96^a^	1390.33 ± 168.29^b^	17.57 ± 1.53^c^
Standard length (cm)	28.16 ± 0.53^a^	44.14 ± 1.98^b^	10.29 ± 0.15^c^
Height (cm)	8.44 ± 0.16^a^	8.82 ± 0.39^b^	2.57 ± 0.03^c^
Condition factor	1.63 ± 0.08	1.58 ± 0.07	1.56 ± 0.08

*Proximate composition*			
Crude protein (% DW)	24.26 ± 0.87^a^	22.57 ± 0.37^b^	21.39 ± 0.15^c^
Crude fat (% DW)	6.64 ± 0.03^a^	7.47 ± 0.02^b^	7.75 ± 0.40^c^
Crude ash (% DW)	1.94 ± 0.02^a^	2.30 ± 0.09^b^	1.59 ± 0.02^c^
Moisture (% WW)	82.40 ± 2.51	85.39 ± 2.36	83.75 ± 2.21

*Mineral composition*			
Macrominerals (mg/g)			
Sodium	0.99 ± 0.00^a^	1.61 ± 0.01^b^	1.61 ± 0.02^c^
Magnesium	1.12 ± 0.01^a^	0.57 ± 0.02^b^	1.15 ± 0.01^c^
Calcium	1.66 ± 0.00^a^	1.55 ± 0.02^b^	1.49 ± 0.04^c^
Potassium	0.71 ± 0.00^a^	0.55 ± 0.02^b^	0.43 ± 0.01^c^
Phosphorous	7.03 ± 0.03^a^	2.74 ± 0.02^b^	6.45 ± 0.07^c^
Microminerals (*μ*g/g)			
Iron	28.30 ± 0.11^a^	28.73 ± 0.08^b^	40.36 ± 0.55^c^
Copper	8.93 ± 0.03^a^	7.46 ± 0.09^b^	6.21 ± 0.32^c^
Manganese	1.64 ± 0.02^a^	2.64 ± 0.02^b^	2.84 ± 0.02^c^
Zinc	24.03 ± 0.45^a^	24.61 ± 0.19^b^	54.46 ± 0.17^c^

Values are *mean*% ± *SE*. Values in the same row followed by different letters are significantly different (*p* < 0.05).

**Table 2:** Fatty acid composition of three species of Bagridae fish.

Fatty acid	*Hemibagrusnemurus*	*Hemibagruswyckii*	*Mystusnigriceps*
C12:0 (lauric acid)	1.97 ± 0.05^a^	3.94 ± 0.01^b^	4.27 ± 0.01^c^
C14:0 (meristic acid)	2.38 ± 0.03^a^	2.72 ± 0.08^b^	2.89 ± 0.01^c^
C16:0 (palmitic acid)	27.23 ± 0.06^a^	30.70 ± 0.06^b^	25.59 ± 0.01^c^
C18:0 (stearic acid)	16.17 ± 0.02^a^	13.54 ± 0.02^b^	10.64 ± 0.01^c^
C20:0 (arachidic acid)	0.23 ± 0.01^a^	0.14 ± 0.01^b^	0.37 ± 0.01^c^
C16:1 (palmitoleic acid)	1.78 ± 0.00^a^	1.27 ± 0.04^b^	4.93 ± 0.01^c^
C18:1(oleic acid)	16.86 ± 0.01^a^	16.18 ± 0.01^b^	16.97 ± 0.01^c^
C18:2 (linoleic acid)	3.24 ± 0.02^a^	3.84 ± 0.33^b^	4.55 ± 0.001^c^
C18:3 (linolenic acid)	1.12 ± 0.01^a^	0.82 ± 0.01^b^	1.57 ± 000^c^
C20:4 (arachidonic acid)	12.13 ± 0.01^a^	12.49 ± 0.09^b^	12.41 ± 0.02^c^
C20:5 (eicosapentaenoic acid; EPA)	2.06 ± 0.08^a^	2.34 ± 0.04^b^	1.35 ± 0.01^c^
C22:6 (docosahexaenoic acid; DHA)	15.65 ± 0.01^a^	11.56 ± 0.04^b^	13.64 ± 0.02^c^

Values are *mean*% ± *SE*. Values in the same row with different superscripts are significantly different (*p* < 0.05).

**Table 3:** Fatty acid *ω*-6/*ω*-3 and polyunsaturated/saturated fatty acid ratio of three species of Bagridae fish.

	*Hemibagrusnemurus*	*Hemibagruswyckii*	*Mystusnigriceps*
*ω*-6/*ω*-3 ratio	0.82 ± 0.00^a^	1.11 ± 0.02^b^	1.02 ± 0.01^c^
P/S ratio	0.69 ± 0.01^a^	0.61 ± 0.06^b^	0.76 ± 0.00^c^
AI	0.87 ± 0.01^a^	0.99 ± 0.06^b^	0.73 ± 0.00^c^
TI	0.40 ± 0.01^a^	0.63 ± 0.01^b^	0.43 ± 0.00^c^

P/S: polyunsaturated/saturated fatty acid; AI: atherogenic index; TI: thrombogenic index. Values are *mean*% ± *SE*. Values in the same row followed by different letters are significantly different (*p* < 0.05).

**Table 4:** Amino acid composition of three species of Bagridae fish.

Amino acid	*Hemibagrusnemurus*	*Hemibagruswyckii*	*Mystusnigriceps*
Aspartic acid	10.39 ± 0.01^a^	9.61 ± 0.04^b^	11.28 ± 0.02^c^
Glutamic acid	24.34 ± 0.00^a^	21.58 ± 0.41^b^	19.29 ± 0.03^c^
Serine	3.74 ± 0.00^a^	5.40 ± 0.03^b^	7.50 ± 0.06^c^
Glycine	4.02 ± 0.00^a^	5.77 ± 0.04^b^	4.69 ± 0.02^c^
Histidine	2.39 ± 0.00^a^	2.90 ± 0.03^b^	2.21 ± 0.02^c^
Arginine	6.17 ± 0.01^a^	8.27 ± 0.03^b^	6.22 ± 0.03^c^
Threonine	4.49 ± 0.01^a^	4.19 ± 0.03^b^	4.39 ± 0.04^c^
Alanine	7.03 ± 0.00^a^	6.04 ± 0.02^b^	7.89 ± 0.01^c^
Proline	2.40 ± 0.00^a^	2.71 ± 0.01^b^	2.21 ± 0.02^c^
Tyrosine	3.91 ± 0.01^a^	3.29 ± 0.06^b^	3.82 ± 0.04^c^
Valine	3.73 ± 0.00^a^	4.02 ± 0.01^b^	3.85 ± 0.04^c^
Methionine	3.42 ± 0.00^a^	1.96 ± 0.02^b^	1.27 ± 0.03^c^
Cystine	0.87 ± 0.00^a^	0.76 ± 0.02^b^	0.75 ± 0.03^c^
Isoleucine	3.82 ± 0.001^a^	3.96 ± 0.003^b^	3.66 ± 0.002^c^
Leucine	6.15 ± 0.000^a^	6.69 ± 0.002^b^	6.28 ± 0.004^c^
Phenylalanine	5.13 ± 0.000^a^	6.03 ± 0.005^b^	5.18 ± 0.027^c^
Lysine	9.61 ± 0.001^a^	9.82 ± 0.026^b^	9.87 ± 0.003^c^

Values are *mean*% ± *SE*. Values in the same row followed by different letters are significantly different (*p* < 0.05).

## Data Availability

The data utilized in this research has not been previously released or published in any form. The datasets employed and/or analysed during the present study can be obtained by contacting the corresponding author.
